# Limited efficacy of a commercial microbial inoculant for improving growth and physiological performance of native plant species

**DOI:** 10.1093/conphys/coae037

**Published:** 2024-06-18

**Authors:** Wei San Wong, Jaume Ruscalleda-Alvarez, Jean W H Yong, Jason C Stevens, Justin M Valliere, Erik J Veneklaas

**Affiliations:** School of Biological Sciences, The University of Western Australia, 35 Stirling Highway, Crawley, WA 6009, Australia; ARC Centre for Mine Site Restoration, School of Molecular and Life Sciences, Curtin University, Kent Street, Bentley, WA 6102, Australia; School of Biological Sciences, The University of Western Australia, 35 Stirling Highway, Crawley, WA 6009, Australia; ARC Centre for Mine Site Restoration, School of Molecular and Life Sciences, Curtin University, Kent Street, Bentley, WA 6102, Australia; School of Biological Sciences, The University of Western Australia, 35 Stirling Highway, Crawley, WA 6009, Australia; ARC Centre for Mine Site Restoration, School of Molecular and Life Sciences, Curtin University, Kent Street, Bentley, WA 6102, Australia; Department of Biosystems and Technology, Swedish University of Agricultural Sciences, Sundsvägen 14, Alnarp, Sweden; School of Biological Sciences, The University of Western Australia, 35 Stirling Highway, Crawley, WA 6009, Australia; ARC Centre for Mine Site Restoration, School of Molecular and Life Sciences, Curtin University, Kent Street, Bentley, WA 6102, Australia; Department of Biodiversity, Conservation and Attractions, Kings Park Science, 1 Kattidj Close, Kings Park, WA 6005, Australia; School of Biological Sciences, The University of Western Australia, 35 Stirling Highway, Crawley, WA 6009, Australia; ARC Centre for Mine Site Restoration, School of Molecular and Life Sciences, Curtin University, Kent Street, Bentley, WA 6102, Australia; Department of Plant Sciences, University of California Davis, Davis, CA 95616, United States; School of Biological Sciences, The University of Western Australia, 35 Stirling Highway, Crawley, WA 6009, Australia; ARC Centre for Mine Site Restoration, School of Molecular and Life Sciences, Curtin University, Kent Street, Bentley, WA 6102, Australia

**Keywords:** Banksia woodlands, microbial inoculation, mine site restoration, phytohormones, xylem sap

## Abstract

Soil microbial inoculants are increasingly being explored as means to improve soil conditions to facilitate ecological restoration. In southwestern Western Australia, highly biodiverse *Banksia* woodland plant communities are increasingly threatened by various factors including climate change, land development and mining. *Banksia* woodland restoration is necessary to conserve this plant community. The use of microbial inoculation in *Banksia* woodland restoration has not yet been investigated. Here, we evaluated the efficacy of a commercial microbial inoculant (GOGO Juice, Neutrog Australia Pty Ltd) for improving the performance of 10 ecologically diverse *Banksia* woodland plant species in a pot experiment. Plants were subjected to one of two watering regimes (well-watered and drought) in combination with microbial inoculation treatments (non-inoculated and inoculated). Plants were maintained under these two watering treatments for 10 weeks, at which point plants in all treatments were subjected to a final drought period lasting 8 weeks. Plant performance was evaluated by plant biomass and allocation, gas exchange parameters, foliar carbon and nitrogen and stable isotope (δ^15^N and δ^13^C) compositions. Plant xylem sap phytohormones were analysed to investigate the effect of microbial inoculation on plant phytohormone profiles and potential relationships with other observed physiological parameters. Across all investigated plant species, inoculation treatments had small effects on plant growth. Further analysis within each species revealed that inoculation treatments did not result in significant biomass gain under well-watered or drought-stressed conditions, and effects on nitrogen nutrition and photosynthesis were variable and minimal. This suggests that the selected commercial microbial inoculant had limited benefits for the tested plant species. Further investigations on the compatibility between the microorganisms (present in the inoculant) and plants, timing of inoculation, viability of the microorganisms and concentration(s) required to achieve effectiveness, under controlled conditions, and field trials are required to test the feasibility and efficacy in actual restoration environments.

## Teaser

The efficacy of a commercial microbial inoculant for improving the performance of 10 Western Australian woodland plant species was evaluated. Across all investigated species, inoculation had small effects on plant growth. Within each species, inoculation did not result in significant biomass gain, and effects on nitrogen nutrition and photosynthesis were variable and minimal.

## Introduction

The southwest Australian floristic region (SWAFR), located in the South West of Western Australia (WA), is one of only two recognized biodiversity hotspots in Australia (Hopper and Gioia, 2004; Ritchie *et al.,* 2021). Situated within the SWAFR is the Swan Coastal Plain dominated by *Banksia* woodland plant communities (Ritchie *et al.,* 2021). These woodlands are dominated by *Banksia* (Proteaceae) trees, and sometimes with scattered *Eucalyptus* and other tree species present within or above the *Banksia* canopy (Commonwealth of Australia, 2016). The understorey has rich plant species, including sclerophyllous shrubs, sedges, rushes and geophytes (Commonwealth of Australia, 2016; Ritchie *et al.,* 2021). These highly biodiverse *Banksia* woodland plant communities have been increasingly threatened by climate change, urbanization, industry and infrastructure development and resource extraction (Rokich, 2016; Ritchie *et al.,* 2021). Throughout the region, sites cleared for mining of silica and building products are legislated for *Banksia* woodland community restoration so as to conserve the diminishing community (DMIRS, 2020). Despite the years of research and groundwork, a lot of challenges remain to be addressed for *Banksia* woodland restoration (Rokich, 2016).

In the close partnership between the Botanic Gardens and Parks Authority (BGPA, WA) and Hanson Australia (construction and building material supplier), various challenges in *Banksia* woodland restoration have been addressed over the years (Rokich, 2016; UDIA, 2019). Some of the challenges addressed are related to substrate handling (soil ripping, fertilizer applications, soil profile reconstruction, soil compaction), weed invasion, seed dormancy and seed loss due to biotic and abiotic factors (Rokich *et al.,* 2000; Golos and Dixon, 2014; Rokich, 2016). Whilst these various research projects over the years have led to improved restoration strategies in this system, *Banksia* woodland restoration remains challenging due to various factors including substrate properties and water availability (drought). Hence, to improve restoration outcomes, new strategies for overcoming these challenges need to be investigated.

In previous research conducted on *Banksia* woodland restoration, little emphasis has been placed on the soil microbial aspects. Soil microorganisms, encompassing bacteria and fungi, are important for multiple ecosystem processes, including nutrient cycling and soil development (Buscot, 2005), and play important roles in plant nutrition, health and stress tolerance (Prakash *et al.,* 2015). Native plant species may also require soil microorganisms as symbiotic partners and/or source of chemical signals essential for their establishment and development (Marschner *et al.,* 2005; Nurfadilah *et al.,* 2013; Lamont *et al.,* 2015). For example, Marschner *et al.* (2005) investigated the bacterial community composition and function of three Banksia species and found differing bacterial community composition between plant species and growth status. Marschner *et al.* (2005) attributed the bacterial community composition to plant species-specific differences in root exudation, highlighting that even closely related plant species may select for different potentially beneficial microorganisms in the soil (Williams and de Vries, 2020). However, little is known about the *Banksia* woodland soil microbial community, the benefits they confer, the mechanisms involved and how this information can be harnessed to improve restoration outcomes.

In sand mining operations, topsoil is stripped and typically stored in stockpiles and later transferred to restoration sites (Rokich *et al.,* 2000). Topsoil disturbance and stockpiling during mining activities alters soil structure, physicochemical characteristics and microbiological properties (Harris *et al.,* 1989; Ghose, 2004). Recent work from Gorzelak *et al.* (2020) found that bacterial richness declined over time in stockpiled topsoils, which could impact plant performance and restoration outcomes. In a study conducted by Birnbaum *et al.* (2017), using soils from the same mine site, *Acacia saligna* seedlings grown in older stockpiles had lower plant biomass and lower root nodule biomass compared to plants grown in younger stockpiles even despite having greater levels of arbuscular mycorrhizal fungi (AMF) colonization. Birnbaum *et al.* (2017) suggested that fungal pathogens could have been the cause of lower plant biomass, but lower plant biomass could also have resulted from photosynthate loss to the large quantity of associated AMF. However, the loss of bacterial richness or keystone species, such as rhizobia essential for the formation of nitrogen-fixing nodules, is also a plausible explanation for reduced plant performance in stockpiled soils (Banerjee *et al.,* 2018; Gorzelak *et al.,* 2020).

Since the mid-1970s, southwest WA has been experiencing dramatic climate change, including increased average temperature and reduced rainfall (Bates *et al.,* 2008; Sudmeyer *et al.,* 2016). Future climate projections include further rises in average temperature, increases in numbers of dry days and reductions in annual precipitation (Sudmeyer *et al.,* 2016). Such changes mean that *Banksia* woodland communities are likely to experience more extreme and frequent stress events such as drought. In fact, drought stress is already a significant challenge in *Banksia* woodland restoration projects. For example, summer drought has been reported to be a primary cause of plant mortality in *Banksia* woodland restoration projects (Brundrett *et al.,* 2020). Therefore, there is a strong need for restoration practices that improve plant stress tolerance and survival in the face of ongoing climate change.

**Table 1:** Plant species investigated in this study

Species	Family	Growth form	Nutrient acquisition strategy	Fire response	Rooting depth
*A. pulchella* R.Br.	Fabaceae	Shrub	NF, AM	Obligate seeder	Deep
*A. cygnorum* Diels subsp. *cygnorum*	Proteaceae	Shrub	CR, NM	Obligate seeder	Deep
*A. fraseriana* (Miq.) L.A.S. Johnson	Casuarinaceae	Tree	AM/ECM, NF	Resprouter	Deep
*A. manglesii* D. Don	Haemodoraceae	Herb	NM	Obligate seeder	Shallow
*B. attenuata* R. Br.	Proteaceae	Tree	NM, CR	Resprouter	Deep
*B. menziesii* R. Br.	Proteaceae	Tree	NM, CR	Resprouter	Deep
*E. todtiana* F. Muell.	Myrtaceae	Tree	AM/ECM	Resprouter	Deep
*H. subvaginata* (Steud.) F. Muell.	Dilleniaceae	Shrub	AM	Obligate seeder	Shallow
*J. floribunda* Endl.	Fabaceae	Shrub	AM	Resprouter	Deep
*K. glabrescens* Toelken	Myrtaceae	Shrub	AM/ECM	Obligate seeder	Shallow

AM, arbuscular mycorrhizal; CR, cluster roots; ECM, ectomycorrhizal; NF, nitrogen fixing; NM, non-mycorrhizal (Pate and Bell, 1999; Brundrett, 2008; Groom and Lamont, 2015; Teste *et al.,* 2020)

The use of microbial inoculants is not a new concept and has been widely used in agriculture, horticulture and forestry (Ruzzi and Aroca, 2015; Santos *et al.,* 2019; Mohan and Rajendran, 2020), and there is increasing interest in applications for ecological restoration (Farrell *et al.,* 2020; Zhong *et al.,* 2023). These inoculants may contain single strains of bacteria or fungi, or a consortia of both (Berg *et al.,* 2021; Canfora *et al.,* 2021). They are reported to confer various benefits on plants, including increased nutrient bioavailability for plant uptake, growth stimulations through phytohormones and increased abiotic stress tolerance via various direct or indirect mechanisms (Glick, 2012; Ruzzi and Aroca, 2015; Oleńska *et al.,* 2020). Plant uptake of phytohormones produced by the microorganisms may also help confer stress tolerance (Ma *et al.,* 2020; Lu *et al.,* 2021). For example, microbial phytohormones can enhance root growth and improve plant water uptake. This is beneficial for the plants, especially seedlings, to survive through summer droughts. Hence, microbial inoculants may have the potential to enhance plant growth, survival and drought tolerance of restored *Banksia* woodlands.

The use of microbial inoculation in *Banksia* woodland restoration has not yet been investigated, and this is the first experiment assessment of a commercial microbial inoculant on *Banksia* woodland plant species. In this pilot study, we evaluated the efficacy of the commercial microbial inoculant GOGO Juice (Neutrog Australia Pty Ltd, a market-leading product sold as a ‘soil and plant probiotic’) for improving the performance of 10 ecologically diverse *Banksia* woodland plant species in a pot study under two watering regimes. We evaluated plant performance using a multifaceted approach, which included measures of growth (i.e. biomass), biomass allocation, photosynthesis and phytohormones. We hypothesized that (1) microbial inoculation treatment would improve plant growth through increased nutrient bioavailability and/or growth stimulation from microbial signals, and (2) inoculated plants would have better stress tolerance and exhibit improved physiological performance under drought conditions compared to non-inoculated individuals, resulting in (3) inoculated plants exhibiting higher concentrations of growth-related phytohormone and lower concentrations of stress-associated phytohormones compared with control (non-inoculated) plants.

## Materials and Methods

### Experimental design

The experiment was conducted in a glasshouse at the University of Western Australia (UWA), Perth, between January and June 2018 for 24 weeks. Potting substrates, consisting of topsoil and overburden (the layer immediately below the topsoil), were sourced from a sand mining operation site of Hanson Australia, situated in the southwest of WA, 50 km northeast of Perth. The area is predominantly *Banksia* woodland; thus, plant species representative of the community with different growth forms, nutrient acquisition strategy and ecological traits were selected as study species (Table 1).

Topsoil and overburden were dried for 18 hours at 50°C in a drying chamber at the UWA Plant Growth Facility to enable the measurement of soil water content from pot weights. Dried topsoil and overburden were sieved through a 2- and 5-mm mechanical sieve, respectively, before use.

Plant seedlings were used in this experiment as some of the species such as *Adenanthos cygnorum* and *Hibbertia subvaginata* are difficult to propagate from seeds (Maher, 2009). Plant seedlings were purchased from local nurseries, namely Natural Area Consulting Management Services (Whiteman, WA; seven species), Plantrite (Bullsbrook, WA; *A. cygnorum* and *Eucalyptus todtiana*) and Apace (North Fremantle, WA; *Anigozanthos manglesii*). All seedlings were uniform in size within each species and received as tubestocks (forestry tube size of 50 mm × 50 mm × 120 mm). Prior to transplanting into substrates collected from the mine site, potting material was carefully removed by washing roots under running water. The individual washed plants were each transplanted immediately into 8 L round plastic pots (250 mm × 235 mm; Garden City Plastics, Forrestfield, WA) lined with transparent plastic bags. Each pot was filled with 6 kg of overburden followed by 3.5 kg of topsoil on top to simulate on-site restoration practices for soil profile reconstruction (average of 50-mm topsoil layer; Rokich, 2016). During the experimental period, the glasshouse was maintained at 24 ± 4°C (day), 18 ± 4°C (night), average relative humidity of 63% and average photosynthetically active radiation (PAR) of 450 μmol m^−2^ s^−1^.

**Figure 1:**
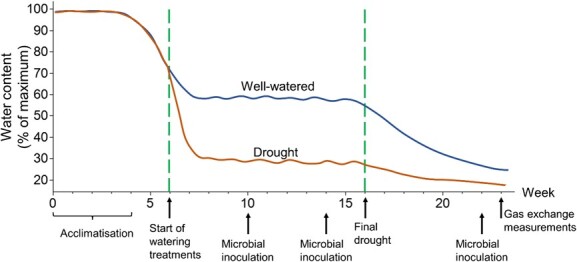
Overview of the experimental timeline, with treatment applications indicated on the X-axis representing time in weeks. Y-axis indicates water content as a percentage of pot water-holding capacity in the well-watered and drought treatment groups. Note that the lines representing the water content are not observed averages but an estimated representation of the experimental conditions.

In this experiment, plants were subjected to one of two watering regimes (well-watered and drought) in combination with microbial inoculation treatments (non-inoculated and inoculated) in a factorial design to determine if microbial inoculation had a beneficial effect on plant drought response. After potting, the plants were acclimatized for 4 weeks at 100% of pot water-holding capacity with two watering sessions each week (Fig. 1). Pot water-holding capacity was determined by weighing pots in their dry state and after saturation with water and subsequent drainage. At this point, the soil held 0.20 g of water per gram of dry soil. During weeks 5–6, watering was reduced to achieve the desired water content levels of 60% and 30% for the well-watered and drought treatments, respectively. These moisture levels were maintained for 10 weeks, with weekly pot weighing and watering to weight. At week 16, all plants were subjected to a final drought treatment (no watering at all) of 8 weeks until the end of the experiment.

**Figure 2:**
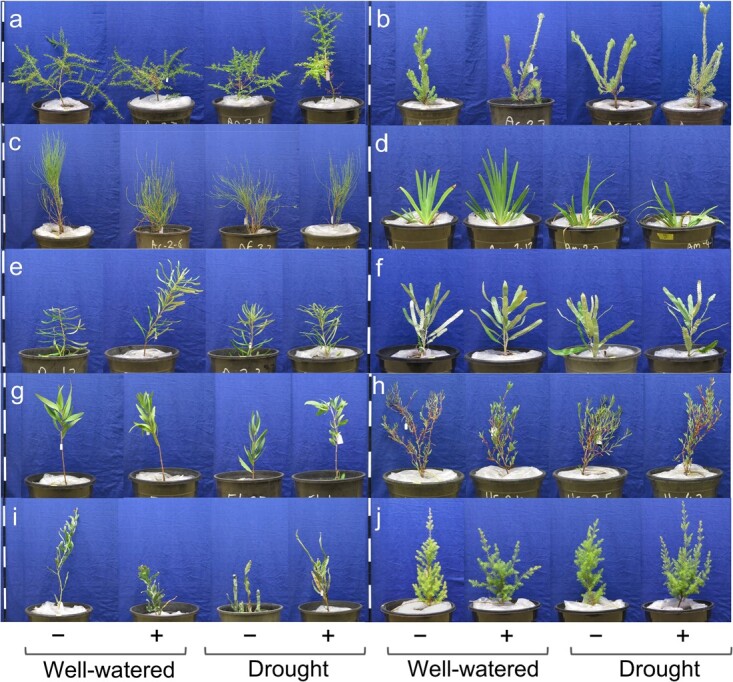
Representative experimental plants of (a) *A*. *pulchella*, (b) *A*. *cygnorum*, (c) *A*. *fraseriana*, (d) *A*. *manglesii*, (e) *B*. *attenuata,* (f) *B*. *menziesii,* (g) *E. todtiana*, (h) *H*. *subvaginata*, (i) *J*. *floribunda* and (j) *K*. *glabrescens*, subjected to well-watered and drought watering treatments and without (−) and with (+) inoculation treatments. Scale bars denote 10-cm intervals.

Microbial inoculant, GOGO Juice (Neutrog^®^ Australia Pty Ltd, Kanmantoo, South Australia; Supplementary Fig. S1), utilized in this experiment was selected from the numerous commercial products available in Australia. It was selected based on its disclosed components, including seaweed extracts, beneficial bacteria (*Pseudomonas*, *Bacillus* and *Azotobacter*), humic/fluvic acid and alginates, which are known to be beneficial for plant growth, stress tolerance and moisture retention (Arioli *et al.,* 2015; Wong *et al.,* 2015). No other products have disclosed their bacterial components. The selected inoculant was used under the assumption that it contained live, viable beneficial microorganisms as stated. The bacterial composition of the GOGO Juice inoculant was verified using 16S rRNA sequencing following the method described in D'Agui *et al.* (2022); Supplementary Table S1). Inoculated plants received three doses of the inoculant (Supplementary Fig. S1) at weeks 10, 14 and 22 to ensure successful inoculation. The last dose was applied during the final drought phase to investigate if inoculation treatment can have beneficial effects on plants under severe drought stress. This inoculant has been previously applied on crop species and Australian native plant species (Frick *et al.,* 2019; Neutrog Australia, 2019; Guymer and Aitchison, 2020). The inoculant was diluted according to manufacturer recommendations (1:100 dilution) and applied to each plant within 20-mm radius at the recommended rate of 5 L/ha. In brief, inoculated plants received 6 ml of diluted inoculant while non-inoculated plants received 6 ml of water. Ten replicates were set up for each treatment group per species and placed in randomized positions on glasshouse benches. The final number of replicates varied between species, ranging between three and six due to plant mortality. Rates of mortality were independent of inoculation. An overview of the experimental timeline with treatment applications indicated is presented in Fig. 1.

**Figure 3:**
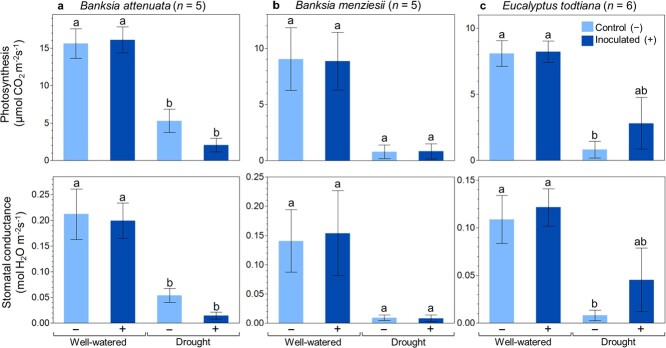
Total biomass of plants grown under well-watered and drought conditions, without (−) and with (+) inoculation treatments. Different letters indicate significant differences between treatments at *P* < 0.05 (from ANOVA with *post hoc* Tukey’s HSD test). Error bars are standard errors.

### Plant physiological measurements

Leaf gas exchange measurements of *Banksia attenuata*, *Banksia menziesii* and *E. todtiana* were taken at week 23 of the experiment, after the plants had been subjected to watering and inoculation treatments, at the end of the final drought period, prior to harvest. In the final 8 weeks prior to these measurements, the plants had not been watered at all. Measurements were made using a portable open system (LI-6400XT, Licor, Lincoln, NE, USA) equipped with the standard leaf chamber, LED light source and carbon dioxide (CO_2_) injector system. All measurements were made in the morning, at PAR of 1500 μmol m^−2^ s^−1^, sample CO_2_ at 385–402 μmol CO_2_ mol^−1^ air and air temperature 20.7–25.5°C. We selected the youngest mature leaf from each plant for these measurements. *B. attenuata* and *B. menziesii* had five replicates, and *E. todtiana* had six replicates, per treatment.

### Xylem sap collection and phytohormone analysis

Phytohormone analyses were conducted on xylem sap of *B. attenuata*, *B. menziesii* and *E. todtiana*, collected pre-dawn prior to the plants’ harvest. These species were selected due to ease of xylem sap harvest, and to elucidate the links between phytohormones and plant physiological processes such as gas exchange, which were measured on these species. During xylem sap collection, plants were cut at approximately 5 cm above soil level and aboveground biomass placed into a pressure chamber (PMS-600; PMS Instrument Company, Albany, OR, USA) for xylem sap collection. The exposed cut stem surfaces were blotted with methanol/formic acid (FA)/water (14:1:2, vol vol^−1^) to inhibit enzymatic degradation of the phytohormones and to remove contaminating cell debris (Noodén *et al.,* 1990). Plant cuttings were placed in a pressure chamber and subjected to increasing pressure until bleeding of xylem sap occurred and then maintained at that constant pressure for sap collection for approximately 5 minutes. The first drops of xylem sap were discarded to avoid contamination. Xylem sap was collected using micropipette and transferred into microcentrifuge tubes kept cold by placing on ice, each containing 50 μl of concentrated FA (Noodén *et al.,* 1990). Xylem sap collected from individual plants ranged between 10 and 500 μl. Collected sap samples were stored in darkness at −80°C until further analysis.

Xylem sap samples within treatment groups per species were pooled (in equal amount) due to varied volume yield from individual plants, which was insufficient for analysis on a per-plant basis. Xylem sap samples were analysed using the same analytical method presented in Wong *et al.* (2022). Briefly, the analysis was performed on an ultra-performance liquid chromatography–electrospray ionization–tandem mass spectrometry (UPLC-ESI-MS/MS, Xevo^®^ TQ-S micro, Waters, Singapore) in ESI-positive (auxins and cytokinins) and ESI-negative [abscisic acid (ABA) and salicylic acid (SA)] mode. The samples were spiked with deuterated standards for 16 hormones (Supplementary Table S2; OlChemIm Ltd, Olomouc, Czech Republic) close to endogenous concentrations (Gosetti *et al.,* 2010) and dried down in a rotary evaporator (Eppendorf Vacufuge plus) at room temperature. The concentrated samples were reconstituted with a starting mobile phase [5% acetonitrile (ACN) and 10% ACN for ESI positive and ESI negative modes, respectively, both with 0.01% FA] for analysis. Samples were analysed at 10× concentration and endogenous concentration in ESI-positive and ESI-negative modes, respectively. Reconstituted samples were analysed in duplicates using an Acquity UPLC^®^ I-Class System equipped with a binary solvent manager, a sample manager with 10-μl loop needle, and an Acquity UPLC^®^ CSH™ C18 column (2.1 × 100 mm, particle size of 1.7 μm) coupled to a triple quadrupole mass spectrometer Xevo^®^ TQ-S micro (Waters, Singapore). The UPLC mobile phase consisted of ACN with 0.01% (vol vol^−1^) FA (A) and water with 0.01% (vol vol^−1^) FA (B), flowing at 0.5 ml min^−1^. System control, data acquisition and data analysis were performed with the MassLynx™ software (version 4.1, Waters, Milford, MA, USA). Phytohormone concentrations were quantified according to the response of the spiked deuterated standards. Results reported are the mean value of duplicate samples that met the criteria of signal-to-noise (S/N) ratio >10. Results with S/N ratio <10 and 3 were deemed below limits of quantification (<LOQ) and below limits of detection (<LOD), respectively. The phytohormones analysed and their respective LOQ and LOD are listed in Supplementary Table S2. Phytohormone analysis on commercial microbial inoculant, GOGO Juice, was attempted but was unsuccessful due to complex matrix effects.

### Biomass and foliar carbon, nitrogen and stable isotopes measurements

Plant growth and biomass allocation were evaluated using biomass sampling at the end of the experiment. Plant biomass was partitioned into shoot and roots. Shoot mass was further split into leaf and stem mass, except for *A. manglesii*, a perennial herb with strap-like leaves that emerge from ground level. Roots were removed from the soil and gently washed to remove attached soil particles. Plant dry mass was determined after drying to a constant weight at 70°C for approximately 72 hours. Leaf mass ratio (leaf mass/total biomass) and root mass ratio (root mass/total biomass) were calculated to determine differences in biomass allocation.

Foliar carbon (C), nitrogen (N) and stable isotopes were analysed for all species except *Jacksonia floribunda* and *Kunzea glabrescens*, which did not show any significant differences in biomass between inoculation treatments in initial analyses. Single, newly mature whole-leaf samples were used for *A. manglesii*, *B. attenuata, B. menziesii* and *E. todtiana*. Multiple newly mature leaves were pooled for *Acacia pulchella*, *A. cygnorum, Allocasuarina fraseriana* and *H. subvaginata*. Samples were oven dried, ground and analysed for δ^15^N and δ^13^C using a continuous flow system consisting of a Delta V Plus mass spectrometer connected with a Thermo Flush 1112 via Conflo IV (Thermo-Finnigan, Germany). All isotopic analyses were performed by the West Australian Biogeochemistry Centre (UWA, Perth).

### Statistical analyses

We first explored the effect of water and inoculation treatments, and the interaction of these factors on measures of plant biomass using two-way analysis of variance (ANOVA), with plant species identity as a random effect. We then conduct three-way ANOVA with the addition of plant species identity as a factor. Plant species showed a significant effect on all parameters, and we followed these analyses with individual two-way ANOVA models by species, with water and inoculation treatments and the interaction of both as fixed effects. For ANOVA models in which significant treatment effects were detected, we utilized Tukey’s HSD tests for *post hoc* mean comparisons.

Comparisons of plant gas exchange parameters, photosynthesis and stomatal conductance, were evaluated using ANOVA followed by *post hoc* Tukey’s HSD tests, performed within each species.

Foliar chemistry and stable isotope data within each species was analysed with two-way ANOVA, with water and inoculation treatments and the interaction of both as fixed effects.

All data were square root or Log normalized to meet assumptions of normal distributions and equal variances. In the event that the data were not able to be normalized, Kruskal–Wallis tests were conducted, followed by Games–Howell *post hoc* test where significant effect tests were detected. All analyses were conducted using JMP^®^ 15.2.0 (SAS Institute Inc.).

Correlations between measured variables on well-watered *B. attenuata*, *B. menziesii* and *E. todtiana* presented in the form of a correlogram were generated by the corrplot package (Wei and Simko, 2017) in the R environment (R Core Team, 2020). Biomass values used for generating the correlogram were normalized within each species by dividing over the mean value. Data from only well-watered treatments were used in generating the correlogram due to incomplete drought treatment data, specifically insufficient phytohormone data for *E. todtiana*.

## Results

### Plant biomass

Across all species, a significant effect of inoculation treatment on plant total biomass (*F* = 4.59, *P* = 0.034) was observed, but not on leaf (*F* = 1.75, *P* = 0.19), stem (*F* = 0.91, *P* = 0.34), root (*F* = 2.74, *P* = 0.10), leaf mass ratio (*F* = 2.83, *P* = 0.09) or root mass ratio (*F* = 0.51, *P* = 0.47), when controlling for plant species identity as a random effect in the statistical model. Averaged across all species, inoculated plants had 8.25% greater biomass at the end of the experiment. Watering treatment had a significant effect on plant total biomass (*F* = 23.70, *P* < 0.001), root mass (*F* = 28.85, *P* < 0.001), leaf mass ratio (*F* = 20.99, *P* < 0.001) and root mass ratio (*F* = 20.16, *P* < 0.001), but not on leaf or stem mass, when controlling for plant species identity as a random effect in the statistical model. Using the same statistical model, the interaction of inoculation and watering treatments did not have significant effect on the measured plant biomass parameters.

As plant species identity had a significant effect on all growth parameters measured (Supplementary Table S3), plant biomass and biomass allocation data were then analysed by species (Table 2, Supplementary Table S4). Overall, we observed few differences in measures of plant biomass and allocation due to inoculation and watering treatments and the interaction, with some exceptions (Supplementary Table S4). There were no statistically significant effects of inoculation treatment on leaf mass for any species. Effects of inoculation treatment were statistically significant on stem mass of *A. pulchella*, and root mass and total biomass of *A. manglesii*. Inoculated *A. pulchella* had higher stem mass, and inoculated *A. manglesii* had higher root mass and total biomass compared to non-inoculated plants (Table 2). Similarly, there were no significant effects of microbial inoculation on biomass allocation except for *A. pulchella*, *A. manglesii* and *E. todtiana* (Supplementary Table S4). Root mass ratio of *A. pulchella* decreased, but it increased in *A. manglesii*, and leaf mass ratio of *A. manglesii* and *E. todtiana* decreased compared to non-inoculated plants (Table 2).

**Table 2:** Plant biomass, partitioned into leaf, stem, root, total biomass, as well as leaf mass ratio and root mass ratio, for plants grown in well-watered and drought conditions, without (−) and with (+) inoculation treatments

		Leaf mass (g)		Stem mass (g)		Root mass (g)		Total biomass (g)		Leaf mass ratio		Root mass ratio	
		Well-watered	Drought	Well-watered	Drought	Well-watered	Drought	Well-watered	Drought	Well-watered	Drought	Well-watered	Drought
*A. pulchella* (*n* = 5)	−	2.55 ± 0.30	1.67 ± 0.15	4.39 ± 0.51^ab^	3.48 ± 0.14^b^	3.11 ± 0.31	4.52 ± 0.66	10.04 ± 0.98	9.66 ± 0.83	0.25 ± 0.05^a^	0.17 ± 0.02^b^	0.31 ± 0.02^b^	0.46 ± 0.03^a^
	+	3.09 ± 0.56	1.97 ± 0.24	6.59 ± 0.89^a^	4.18 ± 0.55^b^	4.45 ± 0.86	3.08 ± 0.45	14.13 ± 2.12	9.23 ± 0.94	0.22 ± 0.02^ab^	0.21 ± 0.02^ab^	0.31 ± 0.03^b^	0.33 ± 0.03^b^
*A. cygnorum* (*n* = 5)	−	4.81 ± 0.55	5.14 ± 0.76	3.98 ± 0.56	3.92 ± 0.77	8.38 ± 1.04^a^	8.07 ± 0.66^ab^	17.17 ± 1.11	17.13 ± 1.03	0.28 ± 0.03	0.30 ± 0.04	0.49 ± 0.06	0.47 ± 0.02
	+	5.51 ± 0.35	5.76 ± 0.72	4.64 ± 0.43	4.38 ± 0.53	12.43 ± 1.67^ab^	6.32 ± 0.66^b^	22.58 ± 1.97	16.46 ± 1.31	0.25 ± 0.03	0.35 ± 0.02	0.54 ± 0.04	0.39 ± 0.04
*A. fraseriana* (*n* = 5)	−	5.26 ± 0.97	4.38 ± 0.45	4.66 ± 0.80	4.91 ± 0.59	8.69 ± 1.67^ab^	5.30 ± 0.58^b^	16.77 ± 2.91	14.59 ± 1.50	0.29 ± 0.03^ab^	0.30 ± 0.02^ab^	0.46 ± 0.03^a^	0.36 ± 0.01^b^
	+	5.24 ± 0.49	5.58 ± 0.42	4.49 ± 1.17	4.56 ± 0.45	11.49 ± 1.29^a^	5.24 ± 0.65^b^	21.22 ± 2.10	15.38 ± 1.33	0.25 ± 0.03^b^	0.36 ± 0.01^a^	0.54 ± 0.02^a^	0.34 ± 0.02^b^
*A. manglesii* (*n* = 5)	−	3.18 ± 0.12	2.89 ± 0.25	−	−	5.84 ± 0.62^a^	4.52 ± 0.25^b^	9.02 ± 0.68	7.40 ± 0.30	0.36 ± 0.02^a^	0.39 ± 0.03^a^	0.64 ± 0.02^b^	0.61 ± 0.03^b^
	+	2.76 ± 0.24	2.67 ± 0.30	−	−	8.96 ± 1.10^b^	5.86 ± 0.47^b^	11.72 ± 1.21	8.53 ± 0.72	0.24 ± 0.02^b^	0.31 ± 0.01^ab^	0.76 ± 0.02^a^	0.69 ± 0.01^ab^
*B. attenuata* (*n* = 5)	−	3.95 ± 0.36	4.28 ± 0.35	0.97 ± 0.14	0.97 ± 0.13	11.76 ± 1.79	10.58 ± 0.53	16.68 ± 2.10	15.83 ± 0.76	0.27 ± 0.01	0.25 ± 0.03	0.70 ± 0.02	0.67 ± 0.02
	+	4.43 ± 0.43	4.33 ± 0.37	1.23 ± 0.16	1.30 ± 0.28	11.23 ± 2.24	14.08 ± 1.84	16.89 ± 2.53	19.70 ± 2.30	0.23 ± 0.02	0.24 ± 0.02	0.69 ± 0.02	0.71 ± 0.02
*B. menziesii* (*n* = 5)	−	4.79 ± 0.71	3.51 ± 0.35	0.95 ± 0.10	0.78 ± 0.11	25.77 ± 2.45^a^	12.69 ± 2.83^b^	31.51 ± 2.94	16.98 ± 2.80	0.15 ± 0.01	0.23 ± 0.05	0.82 ± 0.01	0.72 ± 0.05
	+	4.57 ± 0.31	4.89 ± 0.48	0.94 ± 0.09	0.99 ± 0.06	24.23 ± 2.97^a^	17.54 ± 2.58^ab^	29.75 ± 2.79	23.42 ± 2.87	0.16 ± 0.02	0.22 ± 0.02	0.80 ± 0.03	0.74 ± 0.03
*E. todtiana* (*n* = 6)	−	2.95 ± 0.17	3.19 ± 0.23	1.30 ± 0.14	1.48 ± 0.20	5.33 ± 0.70	5.63 ± 0.29	9.57 ± 0.77	10.50 ± 0.56	0.31 ± 0.02	0.30 ± 0.02	0.55 ± 0.03	0.54 ± 0.01
	+	2.82 ± 0.22	2.90 ± 0.52	1.39 ± 0.18	1.31 ± 0.18	6.76 ± 0.73	5.57 ± 0.50	11.32 ± 0.94	10.19 ± 1.00	0.25 ± 0.02	0.28 ± 0.03	0.59 ± 0.02	0.55 ± 0.03
*H. subvaginata* (*n* = 4)	−	2.31 ± 0.32	3.84 ± 0.22	1.36 ± 0.19	2.15 ± 0.26	3.81 ± 0.33	4.59 ± 0.65	7.48 ± 0.70	10.58 ± 0.89	0.31 ± 0.02	0.37 ± 0.03	0.51 ± 0.03	0.43 ± 0.03
	+	3.61 ± 0.63	2.94 ± 0.12	2.30 ± 0.31	1.97 ± 0.27	4.62 ± 0.29	4.63 ± 0.61	10.52 ± 0.48	9.55 ± 0.56	0.34 ± 0.04	0.31 ± 0.01	0.44 ± 0.03	0.48 ± 0.04
*J. floribunda* (*n* = 3)	−	2.92 ± 0.12	3.26 ± 0.94	1.16 ± 0.28	0.59 ± 0.12	11.51 ± 3.57	8.54 ± 4.9	19.68 ± 3.63	16.24 ± 6.91	0.16 ± 0.03	0.22 ± 0.03	0.56 ± 0.08	0.47 ± 0.08
	+	1.76 ± 0.53	2.65 ± 0.62	0.96 ± 0.41	0.42 ± 0.11	11.24 ± 5.09	7.47 ± 4.40	16.68 ± 3.71	13.60 ± 5.84	0.13 ± 0.05	0.23 ± 0.04	0.61 ± 0.14	0.47 ± 0.10
*K. glabrescens* (*n* = 5)	−	2.96 ± 0.18	3.53 ± 0.36	1.69 ± 0.13	1.77 ± 0.08	8.19 ± 2.12^a^	3.29 ± 0.40^b^	12.84 ± 2.34	8.59 ± 0.79	0.25 ± 0.03^c^	0.41 ± 0.01^a^	0.60 ± 0.05^a^	0.38 ± 0.02^c^
	+	3.24 ± 0.27	3.15 ± 0.27	1.55 ± 0.09	1.46 ± 0.15	6.72 ± 0.99^a^	3.82 ± 0.38^ab^	11.51 ± 1.21	8.42 ± 0.76	0.29 ± 0.02^bc^	0.38 ± 0.01^ab^	0.58 ± 0.03^ab^	0.45 ± 0.01^bc^

Values presented are mean ± standard error. Different letters indicate significant differences between treatments within species at *P* < 0.05 (from ANOVA with *post hoc* Tukey’s HSD test). *A. manglesii* is a perennial herb without true stems; hence, no values are reported for stem mass.

Watering treatment had a significant effect on leaf and stem mass of *A. pulchella*, and root and total biomass mass of *A. cygnorum*, *A. fraseriana*, *A manglesii*, *B. menziesii* and *K. glabrescens*, with well-watered plants having higher biomass than droughted plants (Table 2, Supplementary Table S4). Effects of watering treatment were also statistically significant on the leaf mass ratio of *A. pulchella*, *A. fraseriana, A manglesii, B. menziesii and K. glabrescens*. The same pattern was observed in these species for root mass ratio, except for *B. menziesii*. Leaf mass ratio decreased in *A. pulchella* but increased in *A. fraseriana, A manglesii, B. menziesii and K. glabrescens* under drought treatment. The opposite trend was observed for root mass ratio (Table 2).

The interaction of watering regime and inoculation treatment had a significant effect on the root and total biomass of *A. cygnorum* (Supplementary Table S4). Inoculated *A. cygnorum* under well-watered conditions had the highest root and total biomass followed by non-inoculated plants under well-watered conditions. Regardless of inoculation treatment*, A. cygnorum* in drought treatment had similar root mass, but inoculated plants had higher total biomass (Table 2).

### Plant gas exchange

**Figure 4:**
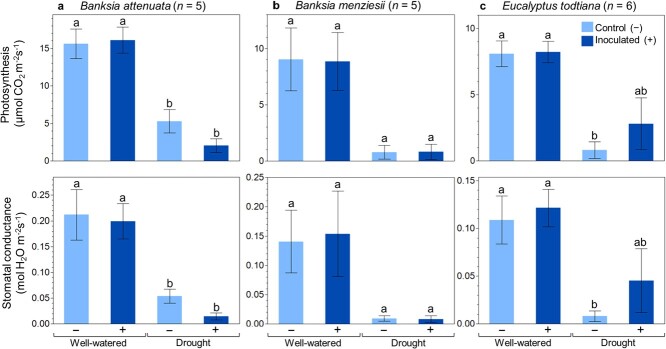
Photosynthesis and stomatal conductance of (a) *B. attenuata*, (b) *B. menziesii* and (c) *E. todtiana* grown under well-watered and drought conditions, without (−) and with (+) inoculation treatments. Measurements were taken at the end of the final drought period. Different letters indicate significant differences between treatments at *P* < 0.05 (from ANOVA with *post hoc* Tukey’s HSD test). Error bars are standard errors.

Photosynthetic rate and stomatal conductance across the three investigated plant species, *B. attenuata*, *B. menziesii* and *E. todtiana*, were varied. Regardless of inoculation treatment, well-watered plants of the three species maintained higher photosynthesis and stomatal conductance (Fig. 4). Whilst inoculation did not cause differences in gas exchange of well-watered plants, we observed contrasting responses under drought: inoculation caused lower photosynthesis and stomatal conductance in *B. attenuata* (Fig. 4a) and higher photosynthesis and stomatal conductance in *E. todtiana* (Fig. 4c). *B. menziesii* did not exhibit an effect of inoculation under drought (Fig. 4b).

### Foliar chemistry and stable isotope composition

Across all investigated species, effects of microbial inoculation on foliar chemistry and stable isotope composition were not statistically significant when controlling for plant species identity as a random effect in statistical models. Using the same statistical analyses, effects of watering regime were statistically significant on foliar stable isotope compositions, δ^13^C (*F* = 8.24, *P* = 0.005) and δ^15^N (*F* = 9.36, *P* = 0.003). Plants under well-watered conditions had lower δ^13^C and δ^15^N values than droughted plants. There were no interactions between inoculation treatment and watering regime.

When plant species identity was included as a fixed effect in the statistical model, together with inoculation treatment and watering regime, plant species identity had a significant impact on foliar chemistry and stable isotope compositions (Supplementary Table S5). Further analyses were therefore conducted on a per-species basis.

Across all investigated species, foliar C content ranged between 390 and 490 mg g^−1^, and minimal differences were observed between treatment groups within each species (Table 3). Effects of microbial inoculation and in interaction with water were statistically significant on foliar C content of *H. subvaginata*, while effects of watering regime were significant for *A. cygnorum* and *A. manglesii* (Supplementary Table S6). In *A. cygnorum*, well-watered plants had higher C content than droughted plants, while *A. manglesii* exhibited the opposite trend. In *H. subvaginata* well-watered non-inoculated plants had the lowest C content, while well-watered inoculated plants had the highest C content, with droughted plants exhibiting intermediate concentrations irrespective of inoculation.

**Table 3:** Foliar carbon (C), nitrogen (N) and stable isotopes δ^13^C and δ^15^N of plants grown in well-watered and drought conditions, without (−) and with (+) inoculation treatments

		C (mg g^−1^)		N (mg g^−1^)	δ^13^C (‰)			δ^15^N (‰)	
		Well-watered	Drought	Well-watered	Drought	Well-watered	Drought	Well-watered	Drought
*A. pulchella* (*n* = 5)	−	450.3 ± 2.1	451.8 ± 3.0	20.9 ± 0.7	23.7 ± 4.9	−32.19 ± 0.45^b^	−30.79 ± 0.11^a^	−1.30 ± 0.26	−0.48 ± 0.11
	+	449.3 ± 4.1	447.0 ± 6.3	21.5 ± 2.8	17.8 ± 2.6	−31.84 ± 0.22^ab^	−31.60 ± 0.11^ab^	−1.06 ± 0.46	−0.74 ± 0.13
*A. cygnorum* (*n* = 5)	−	470.0 ± 3.8^a^	462.4 ± 3.5^ab^	4.5 ± 2.1	3.9 ± 0.9	−32.64 ± 0.29	−31.97 ± 0.29	1.02 ± 0.36	1.18 ± 0.12
	+	472.2 ± 2.6^a^	454.0 ± 2.9^b^	3.1 ± 1.4	1.3 ± 0.4	−32.45 ± 0.19	−31.77 ± 0.47	1.13 ± 0.27	0.44 ± 0.25
*A. fraseriana* (*n* = 5)	−	453.0 ± 0.64	459.3 ± 0.4	3.8 ± 0.8	4.2 ± 0.7	−30.59 ± 0.48	−30.80 ± 0.64	−0.48 ± 0.65	0.17 ± 0.35
	+	460.4 ± 0.5	460.8 ± 1.3	3.8 ± 1.8	3.8 ± 0.9	−31.33 ± 0.18	−30.04 ± 0.15	−1.15 ± 0.83	0.31 ± 0.50
*A. manglesii* (*n* = 5)	−	389.1 ± 6.1	400.6 ± 4.2	3.4 ± 0.7	5.0 ± 1.3	−29.28 ± 0.11	−28.47 ± 0.16	−0.06 ± 0.14	0.50 ± 0.23
	+	393.3 ± 4.4	406.0 ± 2.7	3.4 ± 0.6	4.7 ± 0.2	−29.96 ± 0.54	−29.24 ± 0.38	0.35 ± 0.14	0.27 ± 0.29
*B. attenuata* (*n* = 5)	−	469.4 ± 0.3	466.7 ± 9.8	2.3 ± 0.3	2.9 ± 0.4	−30.56 ± 0.48	−30.59 ± 0.09	0.41 ± 0.26	1.68 ± 0.31
	+	463.7 ± 1.2	475.8 ± 4.3	3.6 ± 0.9	3.1 ± 0.8	−30.62 ± 0.61	−31.07 ± 0.54	0.98 ± 0.22	1.42 ± 0.47
*B. menziesii* (*n* = 5)	−	487.0 ± 4.0	481.1 ± 1.4	3.7 ± 0.9	3.5 ± 1.0	−31.09 ± 0.40	−30.95 ± 0.11	0.75 ± 0.84	1.12 ± 0.67
	+	483.5 ± 6.3	490.4 ± 5.0	3.6 ± 1.3	2.5 ± 0.1	−30.89 ± 0.23	−30.98 ± 0.09	0.78 ± 0.07	1.60 ± 0.21
*E. todtiana* (*n* = 6)	−	481.8 ± 5.2	486.4 ± 4.2	3.5 ± 0.5	2.8 ± 0.6	−30.55 ± 0.53	−30.51 ± 0.63	−3.50 ± 0.33	−2.20 ± 0.46
	+	482.0 ± 4.2	475.9 ± 4.0	2.2 ± 0.2	3.4 ± 0.6	−31.72 ± 0.42	−29.35 ± 1.03	−2.58 ± 0.57	−2.96 ± 0.57
*H. subvaginata* (*n* = 4)	−	399.3 ± 5.9^b^	427.5 ± 3.0^a^	5.0 ± 2.4	4.5 ± 0.7	−32.70 ± 0.27	−31.21 ± 0.41	2.27 ± 0.65	1.87 ± 0.39
	+	432.2 ± 4.7^a^	426.0 ± 7.7^a^	3.2 ± 1.2	4.5 ± 0.9	−31.27 ± 0.38	−32.14 ± 0.92	1.80 ± 0.38	2.41 ± 0.31

Values presented are mean ± standard error. Different letters indicate significant differences between treatments at *P* < 0.05 (from ANOVA with *post hoc* Tukey’s HSD test).

Foliar N concentrations were within the range of 1.5 and 5.0 mg g^−1^ across the investigated species except *A. pulchella* with values at least four times as high (Table 3). Inoculation and watering treatments had no effect on foliar N across all species.

Plant δ^13^C values ranged between −28 and −32**‰**, values which are typical of C3 plants (Marshall *et al.,* 2007). Within species, there were no significant effects of inoculation treatment, but effect of watering regime was significant on *A. pulchella* (Supplementary Table S6). Regardless of inoculation treatment, well-watered *A. pulchella* had more negative δ^13^C values than droughted plants (Table 3).

Plant δ^15^N values varied widely from −3.50 to 2.41‰ (Table 3) between species. Statistical models within each species revealed that there was no significant effect of inoculation treatment in any of the species, but drought generally increased δ^15^N values. Droughted *B. attenuata* had higher positive δ^15^N values than well-watered plants, and similarly, droughted *A. pulchella* and *E. todtiana* had less negative δ^15^N values than well-watered plants (Table 3). In *A. fraseriana*, droughted plants had positive δ^15^N values, whereas well-watered plants had negative values (Table 3).

### Phytohormones

Phytohormones belonging to the class of cytokinins (BAP, BAPR, DHZOG, iPR, *t*ZOG and *t*ZR), ABA and SA were detected in the plant xylem sap samples of all three species investigated, *B. attenuata*, *B. menziesii* and *E. todtiana* (Table 4). Auxins (IAA and IBA) and cytokinins, *c*Z, DHZ, DHZR, iP, K and *t*Z were not detected.

Cytokinins DHZOG and *t*ZOG were observed in much higher concentrations (~1.3× to 12.7×) in *B. menziesii* compared to *B. attenuata* regardless of treatment (Table 4), despite both being species within the same genus. In all three species, iPR was found in higher concentrations in inoculated plants under well-watered conditions compared to plants grown under drought (Table 4).

**Table 4:** Phytohormone concentrations (nmol l^−1^) detected in pooled xylem sap samples

		*B. attenuata* (*n* = 5)		*B. menziesii* (*n* = 5)		*E. todtiana* (*n* = 6)	
		Well-watered	Drought	Well-watered	Drought	Well-watered	Drought
BAP	−	<LOQ	<LOQ	<LOQ	<LOQ	0.124	−
	+	<LOQ	<LOQ	<LOQ	<LOQ	0.118	−
BAPR	−	<LOD	<LOD	<LOD	<LOD	<LOQ	−
	+	<LOD	<LOD	<LOD	<LOD	0.092	−
DHZOG	−	0.608	0.636	3.573	3.409	0.524	−
	+	0.610	0.738	3.091	1.004	4.914	−
iPR	−	0.048	0.066	0.057	0.092	0.107	−
	+	0.072	0.066	0.110	0.060	0.125	−
*t*ZOG	−	4.12	4.82	38.37	29.78	7.15	−
	+	3.06	4.56	38.77	29.17	117.32	−
*t*ZR	−	0.52	0.40	4.66	1.90	0.04	−
	+	1.12	2.17	3.11	0.44	0.15	−
ABA	−	131	67.0	139	90.9	487	14.3
	+	89.9	45.0	69.1	69.2	268	18.9
SA	−	590	583	929	941	62.3	219
	+	598	736	1061	1036	42.9	42.9

Xylem sap samples were pooled from all replicates within each treatment group to yield one sample per species per treatment. <LOD indicates concentration below limits of detection; <LOQ indicates concentration below limits of quantification; − indicates that no samples were available; BAP, N^6^-benzyladenine; BAPR, N^6^-benzyladenosine; DHZOG, dihydrozeatin-*O*-glucoside; iPR, N^6^-isopentenyladenosine; *t*ZOG, *trans*-zeatin-*O*-glucoside; *t*ZR, *trans*-zeatin riboside.

Across all three species, stress-associated phytohormone ABA concentrations were lower in droughted plants compared to well-watered plants. Comparing between inoculation treatments, ABA concentrations were lower in inoculated plants, except drought-treated *E. todtiana* (Table 4). In the well-watered treatment, inoculated plants across all three species had approximately 0.5× lower ABA concentrations than non-inoculated controls. In contrast, in the drought treatment, inoculated *B. attenuata* and *B. menziesii* had 0.7× lower ABA than their non-inoculated controls. Droughted and inoculated *E. todtiana* had 0.3× higher ABA concentration than its non-inoculated control.

Levels of the other stress-associated phytohormone SA showed less variation between treatments in both *B. attenuata* and *B. menziesii*; however, inoculated *E. todtiana* had lower SA than non-inoculated plants in both water treatments (Table 4).

**Figure 5:**
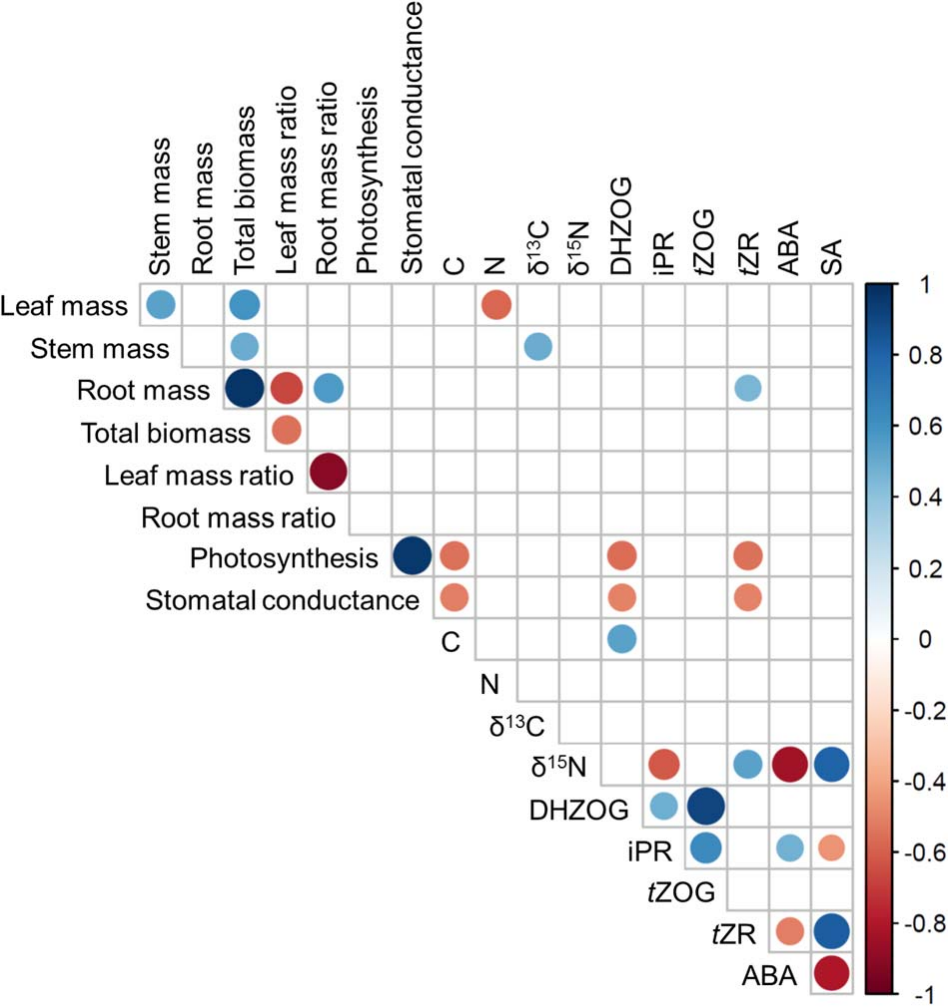
Correlogram for the measured plant growth parameters (leaf, stem, root and total biomass) and resource partitioning (leaf and root mass ratios), gas exchange parameters (photosynthesis and stomatal conductance), foliar chemistry, foliar stable isotope composition (δ^13^C and δ^15^N) and xylem sap phytohormones of *B. attenuata*, *B. menziesii* and *E. todtiana*, grown under well-watered conditions, with and without microbial inoculation. Circle size is proportional to the correlation coefficient. Positive correlation is indicated by blue, while negative correlation is indicated by red. Blank squares indicate that the correlation was not significant (*α* = 0.05)

Correlations between the measured plant growth and physiology parameters and phytohormones in *B. attenuata*, *B. menziesii* and *E. todtiana* were explored via a correlogram (Fig. 5). Cytokinins DHZOG and *t*ZR correlated negatively with gas exchange parameters, photosynthesis and stomatal conductance. However, *t*ZR correlated positively with root mass and total biomass. ABA correlated positively with iPR but negatively with *t*ZR, and the opposite trend occurred for SA.

## Discussion

We explored the effects of a commercial microbial inoculant on the growth and physiological performance of a diverse range of native *Banksia* woodland plant species to evaluate the potential for such products to improve restoration success in post mining sites. The efficacy of this approach was evaluated through various plant responses including growth, physiological adjustments and potential signals in the form of phytohormones. We observed that the beneficial effects of microbial inoculation were minimal, and overall water availability exerted a stronger effect on plant performance. In contrast with our hypotheses, microbial inoculation treatment did not result in significantly enhanced plant growth, and differences in physiological performance between inoculated and non-inoculated plants under drought conditions varied between species. Inoculated plants under well-watered conditions however exhibited a trend of higher iPR concentrations.

Improved plant growth from microbial inoculation treatments has been reported in plant species used for ecological restoration (Schoebitz *et al.,* 2014; Pérez-Fernández *et al.,* 2016; Gonzalez *et al.,* 2018; Solans *et al*., 2021). In contrast, the observed effects of inoculation treatment on plant growth in the native *Banksia* woodland species were minimal. The failure to observe a significant effect of inoculation treatment on plant growth could be due to low product efficacy and/or limitations of the experimental design. Bacterial sequencing of the product (Supplementary Table S1) confirmed the presence of *Bacillus* but not *Pseudomonas* or *Azotobacter*. Bacterial genera *Clostridium*, *Oceanobacillus* and *Virgibacillus* known to be associated with plant growth promotion (Tiwari *et al.,* 2019; Figueiredo *et al.,* 2020; Riahi *et al.,* 2020) were found to be present in the product. It was however not known if the beneficial microorganisms were viable during inoculation. Furthermore, there was no expiration date or production information (e.g. production date or batch number) stated on the product. These results indicate the apparent absence of quality assessments, highlighting the weak regulatory requirements for commercial microbial inoculant products in Australia and even globally (Berg *et al.,* 2020; O’Callaghan *et al.,* 2022).

Overall, our results revealed that water had more significant effects on total plant biomass. Whilst the microbial treatment had a 3-month duration, biomass increase during that period may have been small due to the slow growth rates of these native species (Groom and Lamont, 2015). Therefore, only a relatively small percentage of the final biomass was formed during the experimental period, making it difficult to demonstrate growth benefits and shifts in allocation. Final biomass may also have been differentially affected by biomass loss through shedding of leaves (Chaves *et al.,* 2003), which was not accounted for in this experiment.

One of the potential advantages of microbial inoculation is improved nutrient availability. We did not find evidence for this in the present study. A possible explanation is that the natural *Banksia* woodland substrate is extremely low in nutrients (Rokich *et al.,* 2002), reducing the possibility that introduced microbes cause significantly enhanced nutrient availability for plant uptake. The low nutrient availability is evident in the low N foliar content in both inoculated and non-inoculated plants regardless of water treatment. Furthermore, the seedlings were likely well fertilized during cultivation at the nurseries, providing the plants with surplus nutrients and negating the benefits of microbial inoculation (Porter and Sachs, 2020). We can safely exclude the possibility that the inoculant contained sufficient mineral nutrients to measurably enhance growth: the product contains very low concentrations of nutrients (Supplementary Fig. S1), it was diluted 100 times and only small volumes were applied. This resulted in nutrient additions that were at least 1000 times lower than those applied with recommended doses of common fertilizers for Australian native plants.

In our experiment, the age of the plants (~6 months) may have affected the efficacy of the inoculation treatment (Lopes *et al.,* 2021). Inoculation might have been more effective at a young seedling stage; however, plants were actively growing, and a total of three inoculations were given, which should have provided ample time and opportunity to enhance plant performance. More subsequent inoculations may further enhance efficacy (Bashan, 1986) but are unrealistic for field applications. Also, the number of viable microorganisms present and introduced at each inoculation was unknown. Greater improvements in plant performance may have been achieved with the use of optimal microbial concentrations (Bashan, 1986; Ambrosini *et al.,* 2016; Lance *et al.,* 2019). It has been observed that the use of low inoculant concentrations resulted in low efficacy, while the use of concentrations above optimum resulted in deleterious effects (Bashan, 1986; Lance *et al.,* 2019). In summary, we believe that our experiment should have shown some positive effects if the product had contained sufficient plant performance-enhancing microbes that are compatible with the study species.

Apart from improved plant growth, another benefit of using microbial inoculants is enhanced photosynthetic capacity. This has been widely reported in crop species (Timmusk *et al.,* 2014; Samaniego-Gámez *et al.,* 2016) and in some restoration plant species. In restoration plant species, inoculation treatments increased leaf chlorophyll content (Gonzalez *et al.,* 2018) and helped maintain photosynthetic capacity in drought-stressed plants (Liu *et al.,* 2019). Similarly, in this study, inoculation resulted in enhanced photosynthetic capacity in *E. todtiana* plants subjected to drought treatment. However, an opposite response was observed in inoculated *B. attenuata* under drought conditions, and there was no effect of inoculation on photosynthetic rates in the other species evaluated. These highlight the potential importance of plant-microbial interactions under stressful conditions such as drought, during which soil microorganisms can have positive or negative impacts on the plants (Ulrich *et al.,* 2019). Thus, testing the compatibility between inoculant(s) and targeted plant species before blanket application on restoration site(s) is crucial to avoid wasted expenditure in procuring and applying the inoculant(s). Some species may show no response, as observed in *B. menziesii*, while other species may experience a detrimental impact as exhibited by *B. attenuata*.

Although point measurements of plant photosynthesis and stomatal conductance revealed different responses in all three plant species, foliar δ^13^C, which is often used as a long-term integrative indicator of plant photosynthetic performances (Dawson *et al.,* 2002), revealed little to no effect between inoculated treatments and non-inoculated controls, except in *A. pulchella*. This suggests that the inoculation treatments did not have a long-term impact on the intrinsic photosynthetic capacity of the investigated *Banksia* woodland plant species, with *A. pulchella* being the one exception. The inoculant is claimed to contain *Azotobacter*, which has been reported to form associations with *Acacia* spp. (Sulaiman *et al.,* 2019), which could potentially promote photosynthetic capacity in *A. pulchella* through the production of phytohormones, such as auxins and cytokinins, and through phosphate solubilization (Mrkovacki and Milic, 2001; Ruzzi and Aroca, 2015). However, 16S rRNA sequencing did not confirm the presence of *Azotobacter* in the inoculant (Supplementary Table S1).

The foliar δ^15^N results also further suggest inoculated microorganisms may have interacted with plant species differently, although effects of inoculation on δ^15^N were not statistically significant. The δ^15^N values observed were overall consistent with expected ranges of each species’ nutrient acquisition strategy, non-mycorrhizal (0.9 ± 0.2‰), arbuscular mycorrhizal (−1.1 ± 0.1‰) and ectomycorrhizal (−2.3 ± 0.2‰; Hobbie and Högberg, 2012). These results suggest that most of the mycorrhizal plants had N (^15^N depleted) sourced from their symbiotic mycorrhizal fungi (Hobbie and Högberg, 2012), resulting in negative foliar δ^15^N values. Exceptions in *A. fraseriana*, *A. manglesii* and *H. subvaginata* could be due to complex interactions between plants and microorganisms (fungal and bacterial) and would require further investigations. It cannot be excluded that some of the differences in plant δ^15^N were legacy effects of different substrates and fertilizers used in different nurseries. Interpretations should therefore focus on within-species effects of inoculation and watering. Lower δ^15^N values in non-mycorrhizal species under well-watered conditions may suggest the occurrence of N fixation by free-living microorganisms (Michelsen and Sprent, 1994). However, watering treatments likely impacted soil microorganisms in various direct and indirect ways, resulting in the varied plant responses observed. Under well-watered conditions, indigenous microorganisms in the substrates or microorganisms adhering to the plant roots during transplant may have been established prior to microbial inoculation. These indigenous microorganisms may have then impeded the establishment of inoculated microorganisms (Lopes *et al.,* 2021). Under drought conditions, associations with beneficial microorganisms may increase to help the plants overcome drought stress (Williams and de Vries, 2020).

Results from phytohormone analysis suggest that each plant species may have its own unique phytohormone profile (Osugi and Sakakibara, 2015). However, despite species-specific variation in phytohormone concentrations, there was a general trend of higher concentrations of iPR in inoculated plants under well-watered conditions. iP-type cytokinins are produced by plants and microorganisms, and they are more commonly produced by bacteria (Frébortová and Frébort, 2021). The higher concentrations of iPR in the inoculated plants could have resulted from plant uptake of exogenous cytokinins, such as cytokinins of microbial-origin, and converted into ribosides (e.g. iPR and *t*ZR), which are the transport forms (Dodd *et al.,* 2010; Hluska *et al.,* 2021; Korobova *et al.,* 2021; Lu *et al.,* 2021). Although iPR has lower bioactivity compared to *t*Z and iP (determined by binding affinity to specific receptors), it can be enzymatically converted into more active forms such as iP and *t*Z (Keshishian and Rashotte, 2015; Hluska *et al.,* 2021). Similar with cytokinin ribosides, glycosylated cytokinins DHZOG and *t*ZOG can be enzymatically converted into active forms (Hluska *et al.,* 2021). Although DHZOG and *t*ZOG are generally considered as storage forms, they are also transported in the xylem and have been found to help regulate plant transpiration and leaf senescence (Badenoch-Jones *et al.,* 1996; Hluska *et al.,* 2021).

Cytokinins and ABA are known to interact antagonistically in the regulation of plant developmental processes and responses to abiotic stress, including drought (Großkinsky *et al.,* 2014; Huang *et al.,* 2018). In the *Banksia* woodland species examined here, inoculated plants had higher concentrations of cytokinins but lower ABA concentrations compared to non-inoculated control plants. This indicates that the inoculation treatments might have induced greater drought tolerance in plants. Drought stress may cause shoot cytokinin concentrations to decrease, and inoculation could provide plants with exogenous cytokinins of microbial origin and help delay drought-induced senescence (Verslues, 2016; Hai *et al.,* 2020). Increasing cytokinin concentrations *in planta* can increase plant growth under favourable conditions (Wong *et al.,* 2015) and help improve plant stress resistance via various mechanisms including leaf senescence suppression, maintenance of meristematic activity, and modulation of stress responses (Ogawa *et al.,* 2011; Liu *et al.,* 2013; Veselov *et al.,* 2017; Hallmark and Rashotte, 2020).

In addition to improving plant drought tolerance, the inoculation treatments may have helped reduce the activity or abundance of antagonistic soil biota (e.g. pathogens). Microbial inoculated *E. todtiana* plants had lower SA concentrations compared with control plants. Accumulation of SA is known to be associated with defence response against pathogens and disease (Naidoo *et al.,* 2014). This phenomenon could be further explored and validated in future studies that include measurements of other plant responses, such as the production of secondary metabolites and presence of specific anti-pathogen activity (Naidoo *et al.,* 2014), which usually manifest in response to pathogens. Soil microbial community analysis, which was not conducted in the present study, could also help determine the effects of inoculation treatments on changing the abundance of antagonistic or beneficial soil and root-associated biota.

Whilst the study of correlations between plant performance parameters and phytohormones revealed interesting patterns across the plant species evaluated, further research is needed. Our analyses only included data from well-watered plants, as we were unable to obtain sufficient sample quantities for drought-stressed plants. It is also important to note that phytohormone profiles and activities may differ between plant species, at different developmental stages and environmental conditions (Akhtar *et al*., 2020). This could explain why some phytohormones were not detected or absent in the samples analysed. Furthermore, the list of phytohormones being analysed was not exhaustive. Other forms of phytohormones (e.g. cytokinin-conjugated conjugates with sugars, sugar phosphates and amino acids) may have significant roles in the regulation of developmental processes and stress responses in *Banksia* woodland species (Hoyerová and Hošek, 2020). Despite these limitations, the well-documented ABA–SA antagonism was observed (Cao *et al.,* 2011). Further investigations incorporating a more comprehensive list of phytohormones and their analysis in plant tissues (leaves and roots) would help to elucidate the specific roles of each phytohormone and the cross-talk between different classes. This would improve our understanding of these phytohormones (including microbially originated forms) on plant physiology, particularly for understudied native plants, such as *Banksia* woodland plant species.

## Conclusions

The use of soil microbial inoculants is increasingly explored as means to manipulate soil abiotic and abiotic conditions to improve restoration outcomes (Farrell *et al.,* 2020; Valliere *et al.,* 2020). In this pilot study, application of the selected commercial microbial inoculant on 10 ecologically diverse *Banksia* woodland plant species resulted in few and small benefits for plant performance. These results suggest that the plant responses may be highly species specific, and commercial microbial inoculants may be poorly suited for application on *Banksia* woodland restoration. This study also highlights that much more testing is required before advocating the use of commercial products, typically developed for agricultural/horticultural species, for native plant restoration. Higher numbers of experimental replicates will also be required to identify robust statistical trends in native plant species. Research should include both experimental studies under controlled conditions (as in the present study) and field trials to test feasibility and efficacy in actual restoration environment. More experimental controls such as inactivated microbial inoculum and/or inoculum carrier substance should be included. Important research questions include compatibility between the microorganisms (present in the inoculant) and plants, age of the plants and timing of inoculation, inoculant formulations, viability of the microorganisms in the inoculant, concentration(s) of microorganisms required to achieve effectiveness and the effects of inoculation treatments on root-associated and soil microbial communities (O’Callaghan *et al.,* 2022). It is crucial to test the compatibility between the microorganisms and plants because in the context of ecological restoration, the microorganisms will be introduced to a diversity of plant species, unlike agricultural monoculture applications. Whether generic commercial inoculants will confer sufficient benefits for rehabilitation or restoration remains unknown—a potentially more effective strategy could be to develop site-specific inoculants by culturing microorganisms found in the same or similar environment, such as reference sites (Remke *et al.,* 2020; Solans *et al*., 2021; Gunathunga *et al.,* 2023). Re-establishing soil health by reintroducing and enhancing microbial diversity and biomass has great potential, but inoculation with ineffective, incompatible or even detrimental microbes could be wasteful and counterproductive.

## Supplementary Material

Web_Material_coae037

## Data Availability

The data that support the findings of this study are available from the corresponding authors upon reasonable request.
