# Supplementary material for: Limited efficacy of a commercial microbial inoculant for improving growth and physiological performance of native plant species
Source: Conserv Physiol. 2024 Jun 18;12(1):coae037. doi: 10.1093/conphys/coae037 (PMC11184453; doi:10.1093/conphys/coae037)
Supplement: Web_Material_coae037 [file web_material_coae037.zip › WS_Wong_Suppl Info - revision Apr 2024.pdf]

Supplementary Figure 1. Commercial brochure of microbial inoculant, GOGO Juice (Neutrog® Australia Pty Ltd, Kanmantoo, South Australia), utilised in the experiment. (Source: https://neutrog.com.au/wp-content/uploads/2019/08/NEU-GGJcomm-A4-060519.pdf)

GOGO Juice

About GOGO Juice

GOGO Juice is literally teeming with beneficial micro-biology and is essentially a pro-biotic for your soil and plants.

GOGO Juice combines the "catalystic" power of billions of beneficial bacteria with the well documented benefits of applying kelp, seaweed, fish, coal and lucerne.

Commencing nearly 10 years ago in a small 20 litre anaerobic beer brewing vessel, it has since developed into a series of commercial stainless steel vats, utilising an aerobic brewing system where oxygen and heat are controlled to optimise bacterial activity throughout the 6-8 week brewing period.

The carefully selected microbes have been proliferated utilising complex carbohydrates and sugars through a unique aerobic brewing system in which kelp, seaweed, fish, coal and lucerne have been digested by these bacteria.

Hence, GOGO Juice not only contains a wide variety of bacteria that perform many beneficial functions within the soil, but it also contains numerous other beneficial elements such as natural growth hormones, humic and fulvic acid, amino acids, vitamins and alginates etc (derived from those digested organic compounds), all of which also serve to enhance soil and plant performance.

The resultant product provides a huge boost of the living micro-biology necessary for your soil and plants to perform at their optimum level, increasing their ability to resist pest and disease and to withstand heat stress and frost.

"Just try it" and judge its performance for yourself!

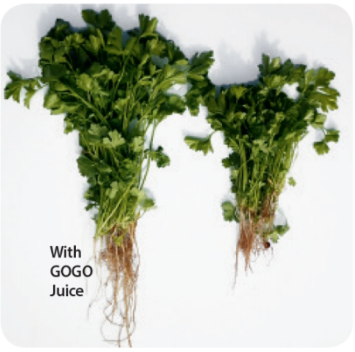

GOGO Juice trial results on parsley at Virginia in South Australia.

GOGO JUICE includes:

Beneficial Bacteria:
Including but not limited to:

- **Pseudomonas** – aids in breaking down organic materials including crop, stubbles and thatch.
- **Bacillus** – aids in unlocking phosphorus.
- **Azotobacter** – aids in nitrogen fixation.

Alginates:
Aid in moisture retention, and consequently in the plant's ability to withstand heat stress and frost.

Humic/Fulvic Acid:
Humic Acid increases nutrient uptake, drought tolerance and seed germination, and Fulvic Acid is in essence, the principal 'active' ingredient in Humic Acid.

Amino Acids:
Building blocks for proteins; important in the metabolic process.

Vitamins:
B1, B2, B3, B6, C, E, Choline, Pantotene, Carotene.

Natural Growth Regulators:

- **Cytokinins** – promote cell division in plant roots and shoots.
- **Auxins** – essential for plant body development. Also co-ordinate many growth and behavioural processes in a plant's life cycle.
- **Triacontanol** – these growth regulators are responsible for promoting root growth and bud production.

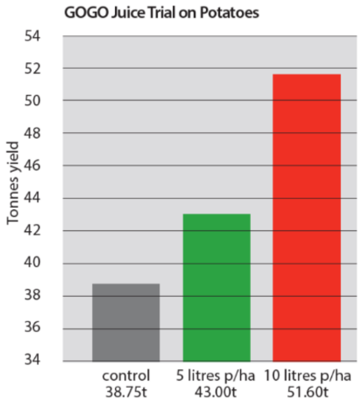

Applications

Apply 5-10 litres per hectare in a minimum dilution of 1:100 (the higher the dilution the better). Lower dilution rates can be utilised if applying to moist soil in the cool of the day. If you have any queries please contact your Neutrog representative.

Apply monthly or as required.

Filtered to 120 microns

Storage and use

GOGO Juice contains live organisms – do not mix with fungicides or bactericides. Store in a cool place, away from direct sunlight below 30 C. Gloves should be worn at all times when handling microbial products. Use diluted product within one (1) week of dilution.

Analysis		%W/V
Nitrogen (N)	as organic	1.00
Phosphorus (P)	as citrate soluble	0.05
Potassium (K)	as organic	0.15
Calcium (Ca)	as organic	0.15
Magnesium (Mg)	as organic	0.07
Sodium (Na)	as organic	0.19
Sulphur (S)	as organic	0.04
Humic Acid	as organic	0.35
		**mg/L**
Boron (B)	as organic	1.00
Copper (Cu)	as organic	2.00
Zinc (Zn)	as organic	1.00
Manganese (Mn)	as organic	12.00
Iron (Fe)	as organic	29.00
Cobalt (Co)	as organic	0.04
Molybdenum (Mo)	as organic	0.50

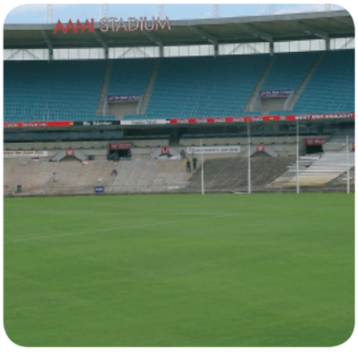

Pooh Bah Club
Join the Pooh Bah Club
Become a member of Neutrog's Pooh Bah Club to receive regular updates on Neutrog, its products and their applications. To join, register your email address at www.neutrog.com.au

Follow us on **facebook** via the link on our website, click the 'like' button and join us for regular updates. All comments, questions, photos and feedback are welcome.
Like

Neutrog Australia Pty Ltd
288 Mine Road, Kanmantoo
South Australia 5252
T (08) 8538 3500
F (08) 8538 3522
E info@neutrog.com.au
W neutrog.com.au

NEUTROG®
Biological Fertilisers

Supplementary Table 1. Bacteria identified in the commercial microbial inoculant using 16S ribosomal RNA sequencing. For details on extraction, sequencing and taxa identification please refer to D'Agui et al. (2022, doi: 10.1111/rec.13738).

**Phylum**	**Class**	**Order**	**Family**	**Genus**
Bacteroidetes	Bacteroidia	Bacteroidales	Porphyromonadaceae	*Paludibacter*
Cyanobacteria	Cyanophyceae			
Firmicutes	Bacilli	Bacillales	Bacillaceae	*Bacillus*
Firmicutes	Bacilli	Bacillales	Bacillaceae	*Oceanobacillus*
Firmicutes	Bacilli	Bacillales	Bacillaceae	*Virgibacillus*
Firmicutes	Bacilli	Bacillales	Thermoactinomycetaceae	*Planifilum*
Firmicutes	Clostridia	Clostridiales	Clostridiaceae	*Clostridium*
Firmicutes	Clostridia	Clostridiales	Lachnospiraceae	*Coprococcus*
Firmicutes	Clostridia	Clostridiales	Peptococcaceae	*Desulfosporosinus*
Firmicutes	Clostridia	Clostridiales	Peptococcaceae	*Desulfotomaculum*
Firmicutes	Clostridia	Clostridiales	Peptococcaceae	*Sporotomaculum*
Firmicutes	Clostridia	Clostridiales	Ruminococcaceae	*Clostridium*
Firmicutes	Clostridia	Clostridiales	Ruminococcaceae	*Ethanoligenens*
Firmicutes	Clostridia	Clostridiales	Ruminococcaceae	*Ruminococcus*
Firmicutes	Clostridia	Clostridiales	Syntrophomonadaceae	*Syntrophomonas*
Firmicutes	Clostridia	Clostridiales	[Tissierellaceae]	*Tepidimicrobium*
WWE1	[Cloacamonae]	[Cloacamonales]	[Cloacamonaceae]	*W22*

Supplementary Table 2. Analytical parameters for the phytohormones and respective deuterated standards.

LOQ: limits of quantification based on S/N ratio <10. LOD: limits of detection based on S/N ratio 3.

Phytohormone class	Analyte	Transition	Cone voltage (V)	Collision voltage (V)	Retention time (min)	LOQ (ng mL^{-1})	LOD (ng mL^{-1})	Deuterated standard	Transition	Cone voltage (V)	Collision voltage (V)	Retention time (min)	Spiked conc. (ng mL^{-1})
Auxins	Indole-3-acetic acid (IAA)	176 > 130	15	20	5.95	0.05	0.025	[$^2H_5$]-IAA	181 > 134	20	25	5.94	0.1
	Indole-3-butyric acid (IBA)	204 > 186	22	15	6.74	0.5	0.025	[$^2H_5$]-IAA	181 > 134	20	25	5.94	0.1
Cytokinins	N^6-Benzyladenine (BAP)	226 > 91	23	22	5.50	0.05	0.025	[$^2H_7$]-BAP	233 > 98	25	24	5.41	0.1
	N^6-Benzyladenosine (BAPR)	358 > 226	10	20	6.07	0.05	0.025	[$^2H_7$]-BAP	233 > 98	25	24	5.41	0.1
	Cis-zeatin (*c*Z)	220 > 136	17	25	1.81	0.25	0.1	[$^2H_5$]-*t*Z	225 > 46	18	21	1.43	0.1
	Dihydrozeatin (DHZ)	222 > 136	20	23	1.56	0.5	0.25	[$^2H_3$]-DHZ	225 > 136	20	26	1.55	0.1
	Dihydrozeatin-O-glucoside (DHZOG)	384 > 222	24	19	1.58	0.5	0.25	[$^2H_3$]-DHZ	225 > 136	20	26	1.55	0.1
	Dihydrozeatin riboside (DHZR)	354 > 136	14	40	3.80	0.05	0.025	[$^2H_3$]-DHZ	225 > 136	20	26	1.55	0.1
	N^6-Isopentenyladenine (iP)	204 > 136	17	17	5.05	0.25	0.025	[$^2H_6$]-iP	210 > 137	20	23	5.01	0.1
	N^6-Isopentenyladenosine (iPR)	336 > 136	22	30	5.99	0.05	0.025	[$^2H_6$]-iP	210 > 137	20	23	5.01	0.1
	Kinetin (K)	216 > 81	20	28	3.39	0.25	0.1	[$^2H_5$]-*t*Z	225 > 46	18	21	1.43	0.1
	Trans-zeatin (*t*Z)	220 > 136	21	19	1.47	0.05	0.025	[$^2H_5$]-*t*Z	225 > 46	18	21	1.43	0.1
	Trans-zeatin-O-glucoside (*t*ZOG)	382 > 220	20	23	1.48	0.5	0.05	[$^2H_5$]-*t*ZOG	387 > 225	17	17	1.41	0.1
	Trans-zeatin riboside (*t*ZR)	352 > 220	14	21	3.60	0.25	0.025	[$^2H_5$]-*t*Z	225 > 46	18	21	1.43	0.1
Others	Abscisic acid (ABA)	263 > 153	20	15	4.94	5	0.25	[$^2H_6$]-ABA	269 > 159	25	16	4.91	10
	Salicylic acid (SA)	137 > 93	25	17	3.92	5	0.1	[$^2H_4$]-SA	141 > 97	25	27	3.88	10

Supplementary Table 3. Analysis of variance results for the effects of plant species, water and inoculation, and their interactions on plant biomass (leaf, stem, root and total), leaf mass ratio and root mass ratio.

Effect	Leaf mass		Stem mass		Root mass		Total biomass		Leaf mass ratio		Root mass ratio	
	F	*p*	F	*p*	F	*p*	F	*p*	F	*p*	F	*p*
Species	25.21	**<0.001**	88.58	**<0.001**	36.79	**<0.001**	34.31	**<0.001**	17.12	**<0.001**	59.72	**<0.001**
Water	0.30	0.584	4.88	**0.029**	30.21	**<0.001**	23.13	**<0.001**	25.25	**<0.001**	26.01	**<0.001**
Species*Water	1.56	0.132	2.45	**0.012**	2.78	**0.005**	1.93	0.051	3.15	**0.002**	5.94	**<0.001**
Inoculation	1.25	0.265	0.75	0.388	1.54	0.216	2.98	0.086	2.35	0.128	0.04	0.845
Species*Inoculation	1.38	0.203	1.59	0.122	0.95	0.481	0.96	0.474	1.89	0.058	1.70	0.095
Water*Inoculation	0.37	0.543	1.62	0.206	2.36	0.127	2.74	0.100	1.10	0.297	0.50	0.479
Species*Water*Inoculation	1.41	0.187	0.63	0.771	1.38	0.203	1.20	0.299	1.46	0.167	1.93	0.052

Supplementary Table 4. Analysis of variance results for the effects of water and inoculation, and the interaction of both on plant biomass (leaf, stem, root and total), leaf mass ratio and root mass ratio. Analysis was performed within each species. Note: *A. manglesii* is a perennial herb without true stems, hence no statistical test values are reported for stem mass.

Species	Effect	Leaf mass		Stem mass		Root mass		Total biomass		Leaf mass ratio		Root mass ratio	
		F	*p*	F	*p*	F	*p*	F	*p*	F	*p*	F	*p*
Acacia pulchella	Water	8.38	**0.011**	9.50	**0.007**	0.01	0.926	3.91	0.066	6.52	**0.021**	9.49	**0.007**
	Inoculation	1.50	0.238	5.99	**0.027**	0.08	0.782	1.63	0.220	0.00	0.990	5.31	**0.035**
	Water*Inoculation	0.12	0.729	1.60	0.225	4.69	**0.046**	2.82	0.113	5.40	**0.034**	4.56	**0.049**
Adenanthos cygnorum	Water	0.22	0.649	0.08	0.787	8.67	**0.010**	4.80	**0.044**	3.01	0.102	4.26	0.056
	Inoculation	1.15	0.300	0.90	0.357	1.12	0.306	2.84	0.112	0.10	0.752	0.14	0.718
	Water*Inoculation	0.00	0.948	0.03	0.865	7.10	**0.017**	4.69	**0.046**	1.35	0.262	2.54	0.131
Allocasuarina fraseriana	Water	0.19	0.672	0.04	0.842	19.56	**<0.001**	5.75	**0.029**	5.93	**0.027**	47.58	**<0.001**
	Inoculation	0.88	0.363	0.11	0.746	1.38	0.257	0.68	0.422	0.30	0.589	1.52	0.236
	Water*Inoculation	0.95	0.345	0.01	0.915	1.61	0.223	0.19	0.665	3.97	0.064	5.59	**0.031**
Anigozanthos manglesii	Water	0.64	0.435	-	-	10.87	**0.005**	9.23	**0.008**	5.45	**0.033**	5.45	**0.033**
	Inoculation	1.80	0.199	-	-	11.44	**0.004**	5.65	**0.030**	20.50	**<0.001**	20.50	**<0.001**
	Water*Inoculation	0.20	0.659	-	-	1.20	0.290	0.74	0.403	0.90	0.356	0.90	0.356
Banksia attenuata	Water	0.09	0.770	0.04	0.853	0.23	0.636	0.23	0.637	0.07	0.794	0.01	0.905
	Inoculation	0.49	0.495	2.39	0.141	0.74	0.404	1.00	0.333	1.20	0.291	0.40	0.537
	Water*Inoculation	0.33	0.576	0.03	0.871	1.37	0.260	0.81	0.383	0.75	0.400	1.16	0.298
Banksia menziesii	Water	0.98	0.338	0.50	0.491	13.26	**0.002**	13.40	**0.002**	5.11	**0.038**	6.22	0.024
	Inoculation	1.43	0.250	1.12	0.306	0.37	0.550	0.67	0.424	0.02	0.901	0.01	0.939
	Water*Inoculation	2.70	0.120	1.40	0.253	1.38	0.257	2.07	0.170	0.03	0.873	0.23	0.638
Eucalyptus todtiana	Water	0.25	0.621	0.10	0.756	0.58	0.454	0.02	0.901	0.16	0.693	0.93	0.346
	Inoculation	0.44	0.515	0.05	0.819	1.38	0.254	0.74	0.399	5.11	**0.035**	1.36	0.258
	Water*Inoculation	0.07	0.791	0.59	0.453	1.64	0.215	1.52	0.232	0.64	0.432	0.35	0.563
Hibbertia subvaginata	Water	1.35	0.267	0.77	0.398	0.63	0.441	2.46	0.143	2.01	0.187	0.42	0.529
	Inoculation	0.28	0.606	2.14	0.169	0.71	0.415	2.20	0.164	1.31	0.279	0.09	0.771
	Water*Inoculation	8.67	0.012	4.50	0.055	0.60	0.455	9.09	**0.011**	0.42	0.532	3.26	0.096
Jacksonia floribunda	Water	0.96	0.356	4.88	0.058	0.55	0.478	0.39	0.549	0.08	0.779	1.34	0.280
	Inoculation	2.01	0.194	0.83	0.389	0.02	0.885	0.29	0.603	4.76	0.061	0.06	0.816
	Water*Inoculation	0.20	0.670	0.00	0.993	0.01	0.932	0.00	0.973	0.25	0.627	0.07	0.804
Kunzea glabrescens	Water	0.76	0.395	0.00	0.959	15.39	**0.001**	6.94	**0.018**	27.82	**<0.001**	30.57	**<0.001**
	Inoculation	0.04	0.851	3.87	0.067	0.03	0.872	0.18	0.676	0.02	0.896	0.52	0.483
	Water*Inoculation	1.43	0.250	0.59	0.452	0.60	0.449	0.09	0.773	2.22	0.156	2.61	0.126

Supplementary Table 5. Analysis of variance results for the effects of plant species, water and inoculation, and their interactions on foliar carbon (C), nitrogen (N), C/N ratio and stable isotopes $\delta^{13}C$ and $\delta^{15}N$ content of plants.

Effect	C		N		C/N		$\delta^{13}C$		$\delta^{15}N$	
	F	*p*	F	*p*	F	*p*	F	*p*	F	*p*
Species	157.26	**<0.001**	78.57	**<0.001**	7.57	**<0.001**	11.61	**<0.001**	57.80	**<0.001**
Water	1.65	0.202	0.01	0.922	0.08	0.780	7.54	**0.008**	7.96	**0.006**
Species*Water	2.49	**0.023**	0.32	0.942	0.53	0.812	0.96	0.470	0.81	0.578
Inoculation	1.65	0.203	2.64	0.108	3.49	0.066	0.21	0.648	0.00	0.965
Species*Inoculation	1.87	0.087	0.52	0.816	1.27	0.279	0.34	0.935	0.17	0.990
Water*Inoculation	1.87	0.176	0.71	0.402	0.05	0.822	0.02	0.888	0.73	0.395
Species*Water*Inoculation	2.54	**0.021**	1.07	0.391	1.33	0.249	2.17	**0.047**	1.23	0.295

Supplementary Table 6. Analysis of variance results for the effects of water and inoculation, and the interaction of both on foliar carbon (C), nitrogen (N) and stable isotopes δ^{13}C and δ^{15}N content. Analysis was performed within each species.

Species	Effect	C		N		δ^{13}C		δ^{15}N	
		F	*p*	F	*p*	F	*p*	F	*p*
Acacia pulchella	Water	0.01	0.939	0.02	0.880	9.95	**0.014**	4.15	0.076
	Inoculation	0.51	0.494	0.61	0.458	0.76	0.409	0.00	0.977
	Water*Inoculation	0.23	0.646	1.19	0.307	4.98	0.056	0.80	0.397
Adenanthos cygnorum	Water	16.22	**0.004**	0.77	0.409	4.25	0.073	1.02	0.343
	Inoculation	0.86	0.380	1.81	0.220	0.34	0.575	1.44	0.265
	Water*Inoculation	2.40	0.160	0.03	0.866	0.00	0.988	2.66	0.142
Allocasuarina fraseriana	Water	0.05	0.838	0.08	0.789	1.68	0.232	2.82	0.132
	Inoculation	0.61	0.461	0.08	0.789	0.00	0.978	0.15	0.712
	Water*Inoculation	0.13	0.734	0.08	0.789	3.24	0.110	0.51	0.497
Anigozanthos manglesii	Water	7.26	**0.027**	3.86	0.085	4.92	0.057	1.31	0.285
	Inoculation	0.99	0.348	0.05	0.833	4.38	0.070	0.18	0.679
	Water*Inoculation	0.01	0.915	0.05	0.833	0.02	0.903	2.34	0.165
Banksia attenuata	Water	0.69	0.421	0.15	0.704	0.27	0.614	6.71	**0.024**
	Inoculation	0.09	0.775	0.32	0.584	0.32	0.582	0.22	0.644
	Water*Inoculation	1.76	0.209	0.63	0.441	0.20	0.665	1.59	0.231
Banksia menziesii	Water	0.01	0.944	0.26	0.621	0.01	0.921	1.18	0.310
	Inoculation	0.33	0.579	0.26	0.621	0.14	0.718	0.21	0.656
	Water*Inoculation	1.69	0.230	0.26	0.621	0.22	0.651	0.17	0.689
Eucalyptus todtiana	Water	0.03	0.869	0.04	0.844	3.04	0.100	0.88	0.361
	Inoculation	1.30	0.272	0.28	0.604	0.00	0.988	0.02	0.877
	Water*Inoculation	1.45	0.246	4.80	**0.044**	2.84	0.111	2.93	0.106
Hibbertia subvaginata	Water	3.81	0.087	0.69	0.431	0.32	0.589	0.06	0.813
	Inoculation	7.73	**0.024**	0.35	0.571	0.21	0.661	0.01	0.937
	Water*Inoculation	9.46	**0.015**	0.35	0.571	4.53	0.066	1.26	0.293
